# Position statement from the British Society of Blood and Marrow Transplantation and Cellular Therapy on insertional oncogenesis in gene‐engineered advanced cell therapy products for treatment of haematological disorders

**DOI:** 10.1111/bjh.70247

**Published:** 2025-11-25

**Authors:** S. Ghorashian, R. Sanderson, J. Lee, A. Black, M. A. O'Reilly, D. I. Marks, J. Snowden, D. Richardson, E. Olavarria

**Affiliations:** ^1^ Department of Haematology Great Ormond Street Hospital for Children London UK; ^2^ Developmental Biology and Cancer UCL‐Great Ormond Street Institute of Child Health London UK; ^3^ Department of Haematology King's College Hospital NHS Foundation Trust London UK; ^4^ British Society of Blood and Marrow Transplantation and Cellular Therapy (BSBMTCT), Guy's Hospital London UK; ^5^ NHS Specialist Pharmacy Service UK; ^6^ Department of Haematology University College London Hospitals NHS Trust London UK; ^7^ Department of Haematology UCL‐Cancer Institute London UK; ^8^ Molecular and Cellular Medicine University of Bristol Bristol UK; ^9^ Department of Haematology Sheffield Teaching Hospitals Sheffield UK; ^10^ University Hospital Southampton Southampton UK; ^11^ Department of Haematology Imperial College Healthcare NHS Trust London UK

**Keywords:** CAR T cell therapy, cell therapy, gene therapy

To the Editor,

In November 2023, the US Food and Drug Administration (FDA) issued a statement on the risk of T‐cell malignancy following chimeric antigen receptor (CAR) T‐cell immunotherapies,^1^ although little mechanistic information or their incidence was available. Thus, the British Society of Blood and Marrow Transplantation and Cellular Therapy (BSBMTCT) recognised the need for a summary of relevant information, presented here, as well as recommendations for the cell therapy community in reporting and investigating such cases.

Gene‐engineered cellular therapies including chimeric antigen (CAR)‐modified T cells (CART) recently gained clinical prominence. Multiple products have now been licensed in advanced haematological malignancies. The recent ZUMA‐7 phase III study showed a clear survival benefit for CART therapy of B cell Non Hodgkin Lymphoma (B‐NHL) in the second line compared to standard of care,^2^ truly representing a paradigm shift in earlier therapy lines. Early phase trials supporting successful correction of inherited haematological disorders with gene‐engineered haematopoietic stem cells (HSCTs) recently culminated in FDA approval of exagamglogene autotemcel and lovotibeglogene autotemcel for the therapy of sickle cell anaemia and FDA/National Institute of Clinical Excellence (NICE) approval of the former for B thalassaemia. Gene therapy of inherited immune and metabolic syndromes as well as the potential of CART therapy of solid organ malignancies have been suggested with tentative breakthrough responses (e.g. Ref [3]).

The occurrence of T‐cell malignancies following therapy with retrovirally‐modified HSCTs for severe combined immunodeficiency highlighted their oncogenic potential. Careful mechanistic investigation^4^ highlighted preferential integration of retrovirally inserted transgenes near the *LMO‐2* gene, triggering modifications to vector design. However, the risk of insertional oncogenesis is not restricted to viral transduction and was associated with gene dysregulation following piggy‐BAC transposon‐based therapy in two patients.^5^

At the time of the FDA statement, 22 cases of T‐cell malignancies were reported following approximately 30 000 US infusions of CART.^6^ Clinical details were available in 14 cases, yet only three malignancies were confirmed to express the CAR transgene, representing 1 in 10 000 events. A subsequent systematic analysis of the FDA adverse event reporting system demonstrated 19 unique cases of T‐cell malignancies reported as a subset of 536 secondary primary malignancies in the context of 12 394 adverse event reports.^7^ Consistent with UK regulatory and accreditation requirements, a need for long‐term (15‐year) follow‐up of patients and addition of this risk to labels for licensed gene‐engineered products was highlighted by the FDA.

Given the uncertainty of post‐marketing reporting to capture true incidence, various groups have also undertaken cohort^8^, ^9^ as well as national studies.^10^ These demonstrated an incidence of second malignancies ranging from 3% to 16% (follow‐up of 2–5 years^11^ of which T‐cell malignancies account for up to 2%).^12^ In some studies, the availability of biopsy material has facilitated confirmation of transgene expression and comparison to a contemporaneous control cohort being treated for haematological malignancies. These studies suggest that the secondary malignancy rate is no higher than for control populations^8^ and confirm the much rarer incidence of true CAR transgene‐associated insertional oncogenesis^13^ which was of the order of 1 in 3000 infusions captured through a national registry,^10^ with a calculated 4‐year cumulative incidence of 0.6%.

A causal relationship to insertional genotoxicity is difficult to establish even in the face of transgene detection within T‐cell lymphoma biopsies, either because of low copy number or other patient risk factors including prior therapy, prolonged immunosuppression and pre‐existing mutations which might also contribute. CAR‐expressing T‐cell malignancies have been reported, for example, following Ciltacabtagene autoleucel.^14^ Here, insertion site and T cell receptor (TCR) sequencing demonstrated a clonal proliferation; however, whole genome/exome analysis also showed *TET2*, *NFKB2, PTPRB* and/or *JAK3* mutations which may have represented pre‐existing clonal haematopoiesis and could have contributed to T‐cell mutational burden.

Clonal haematopoiesis is prevalent in adult patients undergoing CAR T‐cell therapy^15^ and has been associated with both lymphoid^13^ and myeloid malignancies^16^ following CART therapy. In a recent case report of T‐cell lymphoma following tisagenlecleucel infusion for primary central nervous system (CNS) lymphoma,^17^ whole genome sequencing (WGS) of a CAR‐expressing T‐cell lymphoma was undertaken in parallel with WGS of autologous HSCTs, the apheresis product as well as the final CART product. Viral transgene integration analysis of both the CART product and T‐cell lymphoma, as well T‐cell receptor clonality studies on the apheresis, CART product and T‐cell lymphoma were undertaken. With comprehensive characterisation, pre‐existing mutations in *DNMT3A* and *TET2* arising from clonal haematopoietic events rather than insertional oncogenesis were considered causative. A subsequent report of a CAR+ T‐cell lymphoma has highlighted the complex interaction of both germline and somatic mutational status resulting in a subclonal architecture dependent on somatic *TET2* loss of function mutations.^18^ In contrast, in another case, an integration event driving T lymphomagenesis was not associated with clonal haematopoiesis.^10^ Furthermore, it is perhaps surprising that integration events into known tumour suppressor genes have not been more widely evident, although longer follow‐up and systematic reporting/characterisation may be needed. Indeed, the first reported case of mono‐allelic CAR transgene integration resulting in reduced expression of *TP53* was only reported very recently.^19^ In summary, for a better understanding of the mechanisms of oncogenic events following CART therapy, comprehensive genetic characterisation is essential.

It must be noted that non‐malignant expansions of CAR expressing T cells related to insertional disruption of genes such as *TET‐2* and *CBL‐b* have also been reported.^20,21^ Heightened CAR T‐cell expansion in this context was associated with clinical response. As such, diagnostic criteria have been suggested to distinguish T lymphomagenesis from benign CAR T‐cell expansion:

1. Uncontrolled, autonomous proliferation with clinical consequences;
2. Clonal T‐cell expansion on molecular analysis;
3. High mutational burden, with gain‐of‐function mutations in oncogenes or loss‐of‐function mutations in tumour suppressors;
4. Aberrant immunophenotype distinct from that seen in physiological CAR T‐cell persistence;

For those undertaking clinical risk assessment, there are a number of considerations. The population of patients who might benefit from gene‐engineered cellular products ranges from the youngest with inherited disorders to the elderly with malignancy. In surveying nearly 3000 incidences of paediatric/young adult CART delivery,^22^ an international consortium recorded only one case of T‐cell lymphoma in a patient treated with CART for a brain malignancy and pre‐existing constitutional mismatch repair syndrome. There was no evidence of CAR expression and the lymphoma likely arose due to inherited pre‐disposition. In contrast, delivery of lentivirally transduced HSCT therapy for cerebral adrenoleukodystrophy in 67 children was resulted in myeloid malignancies (acute myeloid leukaemia and myelodysplastic syndrome) in seven cases, associated with insertions proximate to MECOM or PRDM16 genes.^23^

Thus, the risk of insertional oncogenesis needs to be set against patient characteristics including age, pre‐existing clonal haematopoiesis, cancer pre‐disposition syndromes and the low but finite risk of a second, unrelated malignancy,^24^ as well as the likelihood of secondary malignancies. This in turn needs to be contextualised against the severity of the disorder being treated and the efficacy of the gene‐engineered product in prolonging life, disease‐free survival or improving its quality.

Although the FDA's statement came as a surprise to the cell therapy community, we hope the information provided here will prove useful to substantiate these concerns, to support effective counselling of patients and facilitate clinical decision‐making around gene‐engineered therapies. Recommendations for reporting and investigation of cases are now available (Supporting Information and through the BSBMTCT website).

References 16–24 are included in the Supporting Information.

## AUTHOR CONTRIBUTIONS

All authors were involved in writing and reviewing the manuscript.

## FUNDING INFORMATION

Not applicable.

## CONFLICT OF INTEREST STATEMENT

SG honoraria from Novartis, trial steering group Autolus, patents UCLB, Autolus. RS Kite/Gilead—Speaker's bureau, honoraria, conference travel, Novartis—Speaker's bureau, honoraria, conference travel, Astrazeneca—Conference travel, adboard, Abbvie—Speaker's bureau. MAO honoraria from Kite Gilead, Novartis and Janssen. Advisory boards Kite Gilead and Autolus. DIM consultancy, Nexcella, Pfizer. JS speaker fees: Janssen; participation on advisory boards for Jazz, BMS, Medac and Vertex; and Independent Data Monitoring Committee membership for a Kiadis Pharma clinical trial. AB, JL, DR, EO—no conflicts of interest.

## ETHICAL APPROVAL STATEMENT

Not applicable.

## Supporting information


Data S1.

## Data Availability

Not applicable.
