# Supplementary material for: Position statement from the British Society of Blood and Marrow Transplantation and Cellular Therapy on insertional oncogenesis in gene‐engineered advanced cell therapy products for treatment of haematological disorders
Source: Br J Haematol. 2025 Nov 25;208(2):776–8. doi: 10.1111/bjh.70247 (PMC12916190; doi:10.1111/bjh.70247)
Supplement: Supplementary file 1 — Data S1. [file BJH-208-776-s001.docx]

**Recommendations:**

1. All patients receiving gene-engineered cellular products should be advised of a very low risk of malignancies arising due to oncogenic mechanisms related to prior clonal haematopoiesis and insertional events, replication competent viral development (where appropriate for the product delivered) as well as general information on safety and efficacy and general risk of new primary / secondary malignancies in relevant patient cohorts
2. All patients should ideally be followed for a minimum of 15 years post infusion with the oversight of a centre specialising in delivery of gene-engineered cellular therapies, although this should be reviewed and may be reduced in future based on true incidence of significant late toxicities e.g. T cell lymphoma related to insertional events
3. All patients developing secondary malignancies following receipt of gene-engineered cellular products should be reported:
   1. To the MHRA Yellow Card reporting scheme
   2. In the relevant EBMT Cellular Therapy Data Collection Form
   3. To the BSBMTCT registry

**Please report the following:**

- **All cases of T-cell malignancies arising following gene-engineered T-cell therapy**
- **All cases of haematological malignancies arising following gene-engineered haematopoietic stem cell therapies**

For this, please email [gst-tr.tnhl_postcart_bsbmtct@nhs.net](mailto:gst-tr.tnhl_postcart_bsbmtct@nhs.net) and provide brief clinical details, a data form will then be sent for completion

- 1. To the market authorisation holders e.g Novartis in the case of tisagenlecleucel

1. All patients developing T-cell malignancies post CAR T-cell infusion should have biopsy material cryopreserved and genomic DNA extracted to enable post hoc genomic analyses e.g. whole genome sequencing, long read DNA sequencing, single cell RNA sequencing where necessary. This can establish relevant mutational burden and enable insertion analysis to be undertaken
2. Related to 4), all patients undergoing engineered cellular therapy should ideally have storage of DNA or failing this, of haematopoietic cells or samples of the apheresis product to enable characterisation of pre-existing mutational burden. Whilst this is ideal, the logistical considerations around routine storage of such material may not be feasible unless such material is also collected as standard of care e.g. to facilitate MRD assessment
3. BSBMTCT will convene an expert advisory panel to advise clinicians and laboratory scientists in the event of development of a malignancy possibly related to insertional oncogenesis
4. A minimum test set in the context of secondary malignancy post infusion of a gene-engineered cellular product should include clonality testing and testing for the inserted transgene

**Complete reference list**

1. FDA Investigating Serious Risk of T-cell Malignancy Following BCMA-Directed or CD19-Directed Autologous Chimeric Antigen Receptor (CAR) T cell Immunotherapies. 2023. Available from: www.fda.gov/vaccines-blood-biologics/safety-availability-biologics/fda-investigating-serious-risk-t-cell-malignancy-following-bcma-directed-or-cd19-directed-autologous

2. Westin JR, Oluwole OO, Kersten MJ, Miklos DB, Perales MA, Ghobadi A, Rapoport AP, Sureda A, Jacobson CA, Farooq U, van Meerten T, Ulrickson M et al. Survival with Axicabtagene Ciloleucel in Large B-Cell Lymphoma. N Engl J Med. 2023;389:148-157.

3. Del Bufalo F, De Angelis B, Caruana I, Del Baldo G, De Ioris MA, Serra A, Mastronuzzi A, Cefalo MG, Pagliara D, Amicucci M, Li Pira G, Leone G et al. GD2-CART01 for Relapsed or Refractory High-Risk Neuroblastoma. N Engl J Med. 2023;388:1284-1295.

4. Howe SJ, Mansour MR, Schwarzwaelder K, Bartholomae C, Hubank M, Kempski H, Brugman MH, Pike-Overzet K, Chatters SJ, de Ridder D, Gilmour KC, Adams S et al. Insertional mutagenesis combined with acquired somatic mutations causes leukemogenesis following gene therapy of SCID-X1 patients. J Clin Invest. 2008;118:3143-3150.

5. Micklethwaite KP, Gowrishankar K, Gloss BS, Li Z, Street JA, Moezzi L, Mach MA, Sutrave G, Clancy LE, Bishop DC, Louie RHY, Cai C et al. Investigation of product-derived lymphoma following infusion of piggyBac-modified CD19 chimeric antigen receptor T cells. Blood. 2021;138:1391-1405.

6. Verdun N, Marks P. Secondary Cancers after Chimeric Antigen Receptor T-Cell Therapy. N Engl J Med. 2024;390:584-586.

7. Elsallab M, Ellithi M, Lunning MA, D’Angelo C, Ma J, Perales MA, Frigault M, Maus MV. Second primary malignancies after commercial CAR T-cell therapy: analysis of the FDA Adverse Events Reporting System. Blood. 2024;143:2099-2105.

8. Steffin DHM, Muhsen IN, Hill LC, Ramos CA, Ahmed N, Hegde M, Wang T, Wu M, Gottschalk S, Whittle SB, Lulla PD, Mamonkin M et al. Long-term follow-up for the development of subsequent malignancies in patients treated with genetically modified IECs. Blood. 2022;140:16-24.

9. Ghilardi G, Fraietta JA, Gerson JN, Van Deerlin VM, Morrissette JJD, Caponetti GC, Paruzzo L, Harris JC, Chong EA, Susanibar Adaniya SP, Svoboda J, Nasta SD et al. T cell lymphoma and secondary primary malignancy risk after commercial CAR T cell therapy. Nat Med. 2024;30:984-989.

10. Dulery R, Guiraud V, Choquet S, Thieblemont C, Bachy E, Barete S, Todesco È, Arnulf B, Boissel N, Baruchel A, Bay J-O, Le Gouill S et al. T cell malignancies after CAR T cell therapy in the DESCAR-T registry. Nat Med. 2025;31:1130-1133.

11. Cappell KM, Kochenderfer JN. Long-term outcomes following CAR T cell therapy: what we know so far. Nat Rev Clin Oncol. 2023;20:359-371.

12. Tix T, Alhomoud M, Shouval R, Cliff ERS, Perales M-A, Cordas Dos Santos DM, Rejeski K. Second Primary Malignancies after CAR T-Cell Therapy: A Systematic Review and Meta-analysis of 5,517 Lymphoma and Myeloma Patients. Clin Cancer Res. 2024;30:4690-4700.

13. Harrison SJ, Nguyen T, Rahman M, Er J, Li J, Li K, Lendvai N, Schecter JM, Banerjee A, Roccia T, Foulk B, Gu J et al. CAR+ T-Cell Lymphoma Post Ciltacabtagene Autoleucel Therapy for Relapsed Refractory Multiple Myeloma. Blood. 2023;142:6939.

14. Miller PG, Sperling AS, Brea EJ, Leick MB, Fell GG, Jan M, Gohil SH, Tai Y-T, Munshi NC, Wu CJ, Neuberg DS, Maus MV et al. Clonal hematopoiesis in patients receiving chimeric antigen receptor T-cell therapy. Blood Adv. 2021;5:2982-2986.

15. Hamilton MP, Sugio T, Noordenbos T, Shi S, Bulterys PL, Liu CL, Kang X, Olsen MN, Good Z, Dahiya S, Frank MJ, Sahaf B et al. Risk of Second Tumors and T-Cell Lymphoma after CAR T-Cell Therapy. N Engl J Med. 2024;390:2047-2060.

16. Vainstein V, Avni B, Grisariu S, Kfir-Erenfeld S, Asherie N, Nachmias B, Auman S, Saban R, Zimran E, Assayag M, Filanovsky K, Horowitz NA et al. Clonal Myeloid Dysplasia Following CAR T-Cell Therapy: Chicken or the Egg? Cancers (Basel). 2023;15:3471.

17. Kobbe G, Brüggemann M, Baermann B-N, Wiegand L, Trautmann H, Yousefian S, Libertini S, Menssen HD, Maier HJ, Ulrich P, Gao J, Bruch P-M et al. Aggressive Lymphoma after CD19 CAR T-Cell Therapy. New England Journal of Medicine. 2024;391:1217-1226.

18. Braun T, Rade M, Merz M, Klepzig H, Große F, Fandrei D, Pham N-N, Kreuz M, Kuhn CK, Kuschel F, Löffler D, Meinel J et al. Multiomic profiling of T cell lymphoma after therapy with anti-BCMA CAR T cells and GPRC5D-directed bispecific antibody. Nat Med. 2025;31:1145-1153.

19. Fraietta JA, Nobles CL, Sammons MA, Lundh S, Carty SA, Reich TJ, Cogdill AP, Morrissette JJD, DeNizio JE, Reddy S, Hwang Y, Gohil M et al. Disruption of TET2 promotes the therapeutic efficacy of CD19-targeted T cells. Nature. 2018;558:307-312.

20. Shah NN, Qin H, Yates B, Su L, Shalabi H, Raffeld M, Ahlman MA, Stetler-Stevenson M, Yuan C, Guo S, Liu S, Hughes SH et al. Clonal expansion of CAR T cells harboring lentivector integration in the CBL gene following anti-CD22 CAR T-cell therapy. Blood Adv. 2019;3:2317-2322.

21. Lamble AJ, Schultz LM, Nguyen K, Hsieh EM, McNerney K, Rouce RH, Gardner RA, Ghorashian S, Shah NN, Maude SL. Risk of T-cell malignancy after CAR T-cell therapy in children, adolescents, and young adults. Blood Adv. 2024;8:3544-3548.

22. Duncan CN, Bledsoe JR, Grzywacz B, Beckman A, Bonner M, Eichler FS, Kühl J-S, Harris MH, Slauson S, Colvin RA, Prasad VK, Downey GF et al. Hematologic Cancer after Gene Therapy for Cerebral Adrenoleukodystrophy. N Engl J Med. 2024;391:1287-1301.

23. Chihara D, Dores GM, Flowers CR, Morton LM. The bidirectional increased risk of B-cell lymphoma and T-cell lymphoma. Blood. 2021;138:785-789.
